# Arsenic and Blood Pressure: A Long-Term Relationship

**DOI:** 10.1289/ehp.123-A218

**Published:** 2015-08-01

**Authors:** Nate Seltenrich

**Affiliations:** Nate Seltenrich covers science and the environment from Petaluma, CA. His work has appeared in *High Country News*, *Sierra*, *Yale Environment 360*, *Earth Island Journal*, and other regional and national publications.

Overexposure to naturally occurring arsenic in groundwater and soil can cause a variety of cancers and has been associated with developmental effects, neurotoxicity, diabetes, and cardiovascular disease.^1^ In this issue of *EHP*, researchers provide new evidence of arsenic’s ability to elevate blood pressure, potentially leading to hypertension and more serious clinical outcomes.^2^

Globally, 200 million people are estimated to drink water exposing them to arsenic at concentrations above the World Health Organization’s recommended limit of 10 µg/L.^3^ An estimated 35–77 million of these people reside in Bangladesh alone,^4^^,^^5^ where nearly all rural households rely on groundwater for drinking water.^6^

**Figure:**
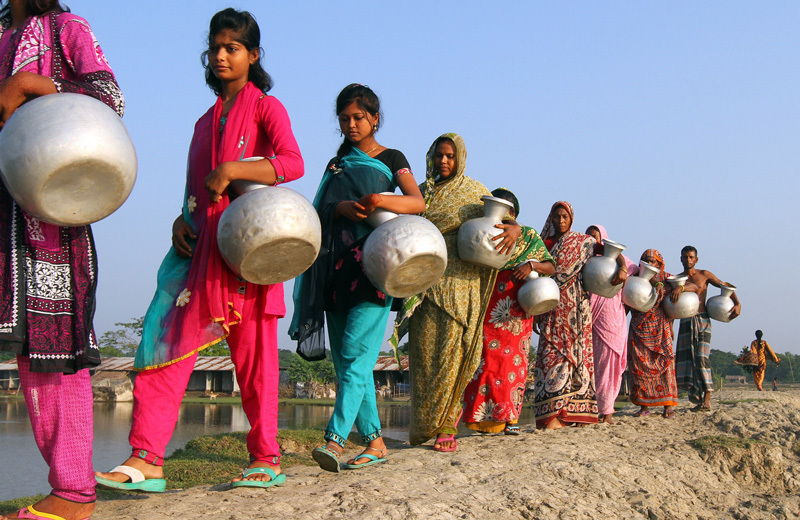
Widespread arsenic exposure via drinking water in Bangladesh has been called the “largest mass poisoning of a population in history.”^10^ © SK Hasan Ali/Demotix/Corbis

To gain insight into the link between arsenic exposure and changes in blood pressure, researchers based at the University of Chicago, Columbia University, and New York Langone Medical Center analyzed data for 10,853 individuals participating in the Health Effects of Arsenic Longitudinal Study (HEALS), a long-term prospective cohort study. These individuals were exposed to low to moderately high levels of arsenic from nearly 6,000 contaminated wells in an area of Bangladesh outside the capital of Dhaka.^7^ (Senior author Yu Chen, an associate professor of population health at New York University, says the cohort has since been expanded to cover a wider area, including data from 11,000 wells.)

“We are trying to reveal the mechanisms that link arsenic exposure and cardiovascular disease,” says first author Jieying Jiang. “We think that increasing blood pressure level might be one of those mechanisms.” Other potential mechanisms include atherosclerosis, oxidative stress, inflammation, and vascular stiffness, which are interrelated and associated with blood pressure, Chen notes.

“The issue with arsenic and hypertension has always been a little more controversial than other outcomes,” says Ana Navas-Acien, coauthor of a 2012 review of the subject.^8^ She explains that many earlier studies were cross-sectional in design, meaning arsenic exposure and incidence of hypertension were examined at a single point in time, so observed associations were harder to interpret. “If you look at our systematic review, you can see there was a tendency for saying that yes, there is an association, but it was not a clear yes,” she says. “This study definitely clarifies that [association]. It shows it’s extremely important to have prospective data.”

Working with a field staff of more than 100 people, the authors of the current study analyzed blood pressure readings taken from each cohort member four times between October 2000 and March 2009, with the initial measurements serving as a baseline. To assess arsenic exposure, they tested well water and urine samples collected at each of the four visits.

The results showed positive associations between arsenic exposure, whether measured in well water or urine samples, and annual increases in systolic and diastolic blood pressure. These associations were stronger among participants who were oldest at baseline.^2^

Even when controlling for age, sex, smoking status, educational status, and diabetes history, the researchers saw an average annual increase in systolic blood pressure of 0.43 mmHg for participants exposed to medium-low levels of arsenic in groundwater (12–62 µg/L), 0.54 mmHg for those exposed to medium-high levels (62–148 µg/L), and 0.48 mmHg for those exposed to high levels (above 148 µg/L), as compared with the control group, which was exposed to levels below 12 µg/L. The authors speculate that baseline blood pressure could have already been affected significantly by past arsenic exposure, such that only a limited increase could be further observed.^2^

The results thus reveal a nonmonotonic dose response, in which individuals exposed to the highest arsenic levels in the study often had a smaller increase than those exposed to medium-high levels.^2^ *In vitro* research has shown arsenic to have a nonlinear effect on blood vessel formation and tumor growth.^9^

In any case, while the observed increases are moderate enough that they don’t point directly to clinical outcomes at the individual level, Navas-Acien says that across a broadly exposed population such as Bangladesh’s, they could translate to more people with hypertension and cardiovascular disease. “If even a small portion of cardiovascular disease can be due to arsenic,” Chen agrees, “that may mean a lot of numbers.”
